# Indolethylamine-N-Methyltransferase Inhibits Proliferation and Promotes Apoptosis of Human Prostate Cancer Cells: A Mechanistic Exploration

**DOI:** 10.3389/fcell.2022.805402

**Published:** 2022-02-17

**Authors:** Wang Jianfeng, Wang Yutao, Bi Jianbin

**Affiliations:** Department of Urology, The First Hospital of China Medical University, Shenyang, China

**Keywords:** indolethylamine-N-methyltransferase, proliferation, apoptosis, prostate cancer, mechanism

## Abstract

Indolethylamine-N-methyltransferase (INMT) is a methyltransferase downregulated in lung cancer, meningioma, and prostate cancer; however, its role and mechanism in prostate cancer remain unclear. By analyzing The Cancer Genome Atlas (TCGA)-PRAD, we found that the expression of INMT in prostate cancer was lower than that of adjacent non-cancerous prostate tissues and was significantly correlated with lymph node metastasis Gleason score, PSA expression, and survival. Combined with the GSE46602 cohorts for pathway enrichment analysis, we found that INMT was involved in regulating the MAPK, TGFβ, and Wnt signaling pathways. After overexpression of INMT in prostate cancer cell lines 22Rv1 and PC-3, we found an effect of INMT on these tumor signal pathways; overexpression of INMT inhibited the proliferation of prostate cancer cells and promoted apoptosis. Using the ESTIMATE algorithm, we found that with the increase of INMT expression, immune and stromal scores in the tumor microenvironment increased, immune response intensity increased, and tumor purity decreased. The difference in INMT expression affected the proportion of several immune cells. According to PRISM and CTRP2.0, the potential therapeutic agents associated with the INMT expression subgroup in TCGA were predicted. The area under the curve (AUC) values of 26 compounds positively correlated with the expression of INMT, while the AUC values of 14 compounds were negatively correlated with the expression of INMT. These findings suggest that INMT may affect prostate cancer’s occurrence, development, and drug sensitivity via various tumor signaling pathways and tumor microenvironments.

## Introduction

Prostate cancer is the most common cancer in men; there are 1.1 million newly diagnosed cases of prostate cancer in the world every year. About 10% (307,000 cases) of male cancer-related deaths are caused by prostate cancer (Flores-Fraile et al., 2020; Woodcock et al., 2020). Between 2003 and 2017, the overall incidence rate of prostate cancer decreased; however, the proportion of patients diagnosed with metastatic prostate cancer increased from 4 to 8% (Siegel et al., 2020). In recent years, the outcomes of prostate cancer, especially localized prostate cancer, have improved; however, the 5-years relative survival rate of patients with metastatic prostate cancer remained low (Liu et al., 2012; Ihle et al., 2019). Therefore, the research and development of biomarkers associated with prostate cancer metastasis, including prostate-specific antigen (PSA), androgen receptor splice variant 7 (ARV*7*), and transforming growth factor-beta (TGFβ), might guide the early treatment decision of tumor metastasis and significantly improve outcomes (Kretschmer and Tilki, 2017; Manca et al., 2017; Bastos and Antonarakis, 2018).

Indolethylamine-N-methyltransferase (INMT) is a methyltransferase that regulates the N-methylation of tryptamine family proteins (Chu et al., 2014; Torres et al., 2019). It is associated with the development and activity of the nervous system and participates in the detoxification of selenium compounds (Kim et al., 2015; Kuehnelt et al., 2015). Studies suggested that the expression of INMT is downregulated in lung cancer, meningioma, and prostate cancer (Kopantzev et al., 2008; Larkin et al., 2012; Schulten et al., 2016); however, its role and mechanism in cancer, especially prostate cancer, remain unclear. The study of its molecular mechanism may help deepen the understanding of tumorigenesis and development and find new targets in various cancers.

In the present study, we analyzed The Cancer Genome Atlas (TCGA)-PRAD and found that the expression of INMT in prostate cancer was lower than that of adjacent non-cancerous prostate tissues. INMT significantly correlated with lymph node metastasis, Gleason score, PSA expression, and survival. Pathway enrichment analysis was performed in combination with GSE46602 cohorts. After overexpression of INMT in prostate cancer cell lines 22Rv1 and PC-3, we confirmed that INMT inhibited MAPK, TGFβ, and Wnt signaling pathways, inhibited the proliferation of prostate cancer cells, and promoted apoptosis. We found that the expression of INMT correlated with tumor microenvironment and immune cell infiltration using the ESTIMATE algorithm. According to PRISM and CTRP2.0, the potential therapeutic agents associated with the INMT expression subgroup in TCGA were predicted. These findings INMT may affect the occurrence, development, and drug sensitivity of prostate cancer via various tumor signaling pathways and tumor microenvironments.

## Results

### The Expression and Clinical Value of INMT in Prostate Cancer

**FIGURE 1:**
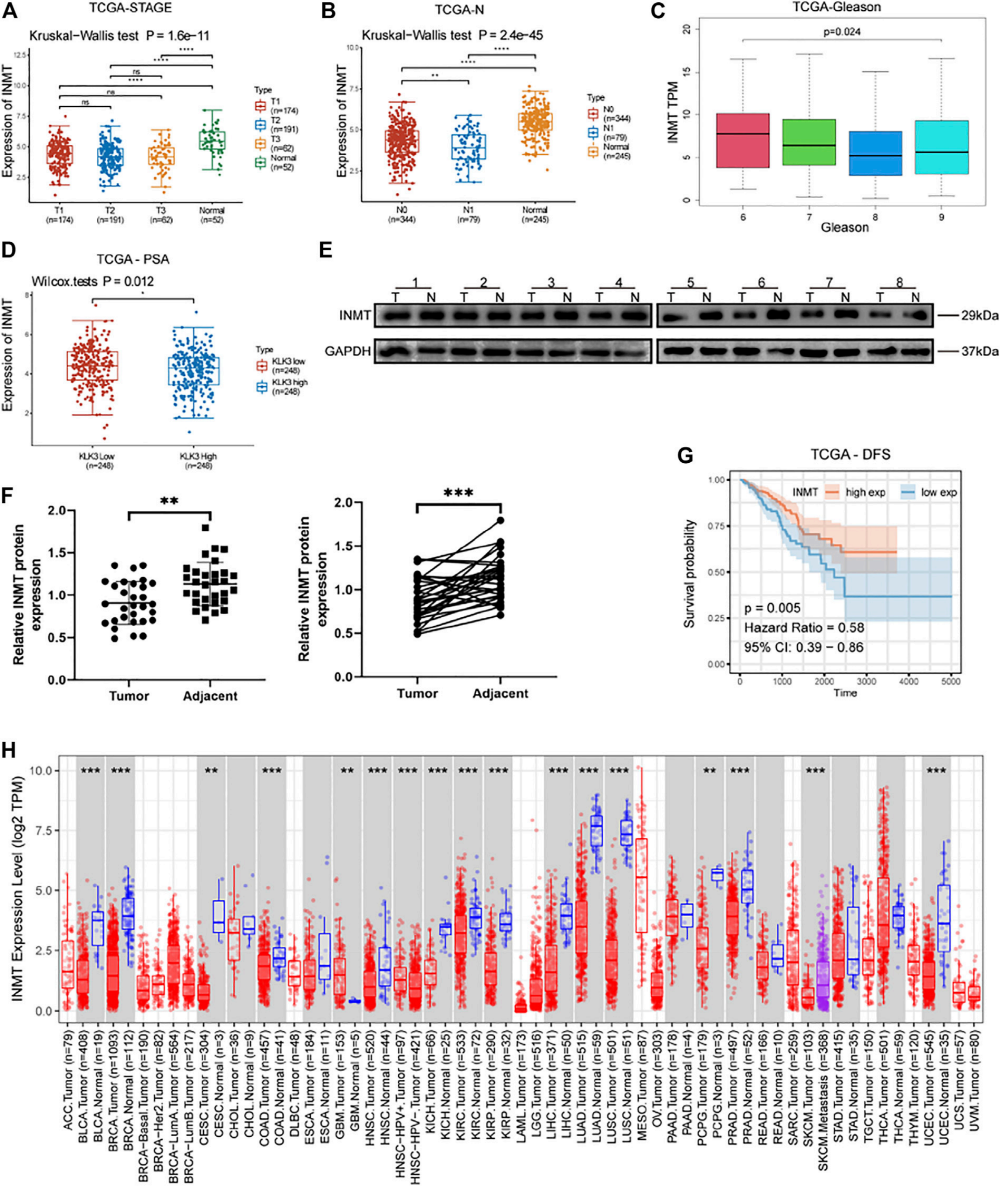
**(A)** Expression of INMT in three clinical stages and normal prostate tissues of TCGA-PRAD (*p* = 1.6e−11), Tx classification according to TNM stage of prostate cancer (AJCC, 2017). **(B)** The expression of INMT in various lymph node metastasis groups and normal tissues of TCGA-PRAD (*p* = 2.4e−45). **(C)** The expression of INMT in various Gleason score groups of TCGA-PRAD (*p* < 0.001). **(D)** The expression of INMT in the high PSA and low PSA groups of TCGA-PRAD (*p* = 0.012). **(E,F)** The expression of INMT protein in 30 pairs of clinical samples of prostate cancer (T) and adjacent non-cancerous prostate tissues (N) (*p* < 0.001). **(G)** Using survival analysis, we found that the disease-free survival time in the INMT high-expression group was significantly longer than in the INMT low-expression group in TCGA-PRAD (*p* = 0.005, Hazard Ratio = 0.58). **(H)** Pan-cancer analysis of the TIMER database showing the expression level of INMT in cancer tissues.

By analyzing TCGA-PRAD, we found that the RNA expression of INMT was remarkably lower in diverse stages of prostate cancer in contrast with the adjacent non-cancerous prostate tissues (Figure 1A, *p* < 0.001). Kruskal−Wallis test and clinical correlation assessment were carried out on TCGA-PRAD and exhibited that the RNA expression of INMT in prostate cancer with lymph node metastasis was significantly lower than that of patients without lymph node metastasis (Figure 1B, *p* < 0.01). With the increase of the Gleason score, the RNA expression level of INMT decreased (Figure 1C, *p* < 0.001). The RNA expression of INMT in the PSA (*KLK3*) high-expression subgroup was significantly lower than that of the PSA low-expression subgroup (Figure 1D, *p* = 0.012). To confirm the difference in the transcription level of INMT in prostate cancer and adjacent non-cancerous prostate tissues analyzed in TCGA, we used western blotting to assess the levels of INMT protein in 30 pairs of prostate cancer and adjacent non-cancerous prostate tissues. Congruent with TCGA, the content of INMT protein in the prostate cancer tissues was reduced, in contrast with the adjacent non-cancerous prostate tissues (Figures 1E,F, *p* < 0.01). The baseline and the pathological data for 30 individuals with prostate cancer are given in Supplementary Table 1. In survival assessment with disease-free survival as the endpoint, the survival benefit of patients exhibiting high RNA expression of INMT was better in contrast with that of those who had low RNA expression of INMT (Figure 1G, *p* = 0.005). To investigate whether the low expression of INMT is unique to prostate cancer or the universality of cancer, we have used pan-cancer exploration of the Tumor Immune Estimation Resource (TIMER) data resource to show that the RNA expression of INMT was lower in most cancer tissues, such as bladder urothelial carcinoma, breast invasive carcinoma, ceramic square cell carcinoma and endocervical adenocarcinoma, colon adenocarcinoma, and head and neck squamous cell carcinoma, and kidney chromophobe (Figure 1H). This suggests that low expression of INMT may be a common phenomenon in cancer cells.

### Overexpression of INMT Affects MAPK, TGFβ, and Wnt Signaling Pathways

**FIGURE 2:**
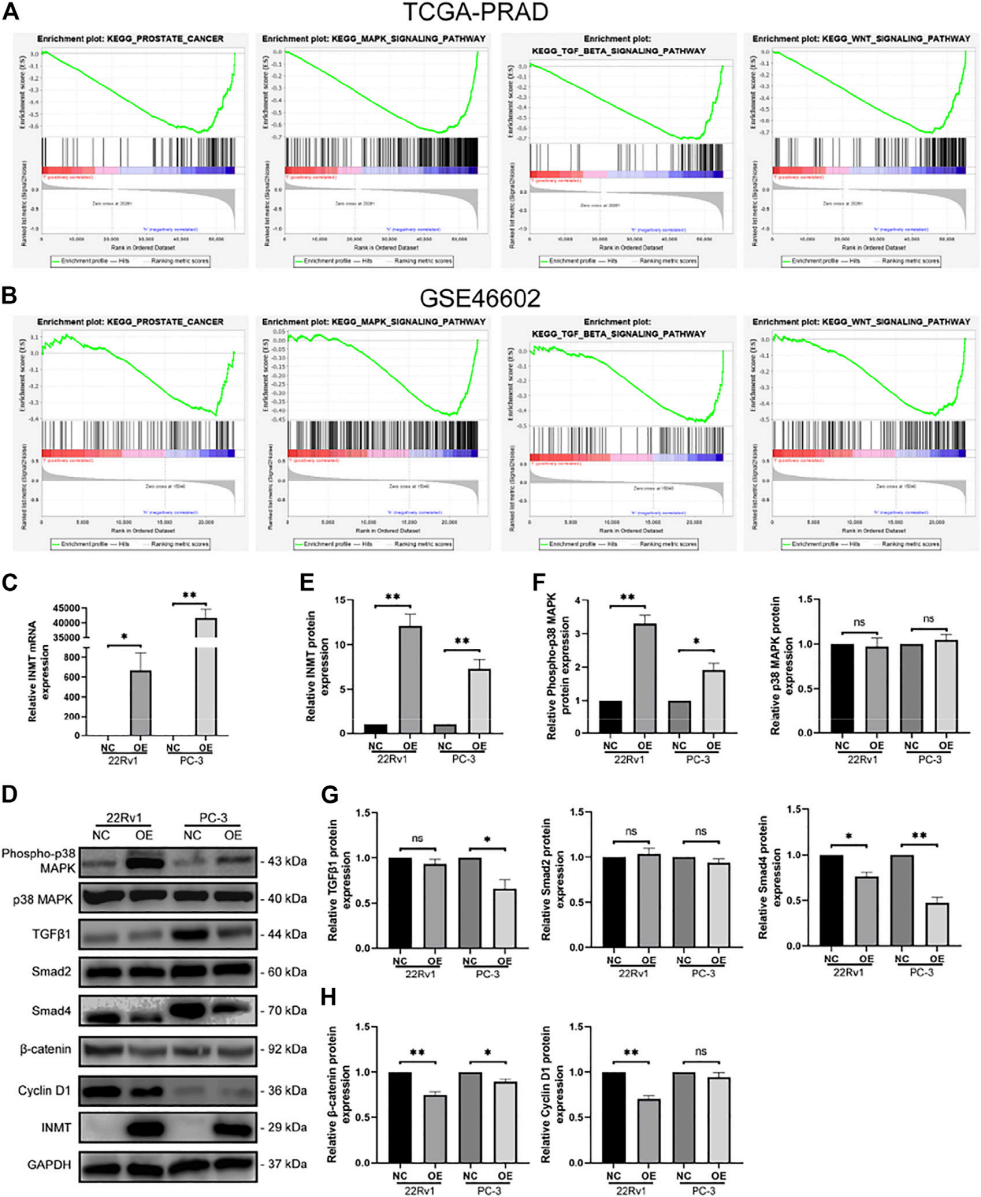
Correlation between INMT and tumor signaling pathways. **(A,B)** In TCGA and GSE46602, INMT was enriched in prostate cancer, MAPK signaling, TGFβ signaling, and Wnt signaling pathways. After transfection with corresponding negative control lentiviral vector and INMT overexpression lentiviral vector in 22Rv1 and PC-3, **(C)** mRNA levels of INMT were measured using real-time PCR, and **(D,E)** protein levels of INMT were measured using western blot. **(D,F)** Protein levels of MAPK signaling pathway-related molecules (Phospho-p38 MAPK, p38 MAPK) were measured using western blotting. **(D,G)** Protein levels of TGFβ signaling pathway-related molecules (TGFβ1, Smad2, and Smad4) were measured using western blot. (D and H) The protein levels of Wnt/*β*-catenin signaling pathway-related molecules (*β-*catenin and cyclin D1) were measured using western blot. The samples derive from the same experiment, and those blots were processed in parallel.

We performed pathway enrichment analysis on TCGA-PRAD and GSE46602 cohorts and found that INMT was closely associated with prostate cancer progression and was involved in regulating the MAPK, TGFβ, and Wnt signaling cascades (Figures 2A,B). To verify the effect of INMT on these pathways, we transfected hormone-sensitive prostate cancer cells (22Rv1) and hormone-resistant prostate cancer cells (PC-3) with INMT overexpression lentiviral vector or the corresponding negative control lentiviral vector. RT-PCR, along with the western blotting results, exhibited that the transcript and protein content of INMT in the overexpression (OE) group were remarkably higher in contrast with that in the negative control (NC) group (Figures 2C–E). Western blotting data exhibited that phospho-p38 MAPK was remarkably upregulated after overexpression of INMT; however, the total amount of p38 MAPK did not change significantly (Figures 2D,F). In contrast with the NC group, the expression of Smad4, a critical factor in the TGFβ signaling pathway, decreased significantly in the OE group. Only in the PC-3 cell line, the expression of TGFβ1 decreased due to the overexpression of INMT (Figures 2D,G). The critical factors in the Wnt signaling cascade (cyclin D1 and *β*-catenin) were downregulated in the OE group compared to the NC group (Figures 2D,H).

### INMT Co-expression Network and Biological Function Analysis

**FIGURE 3:**
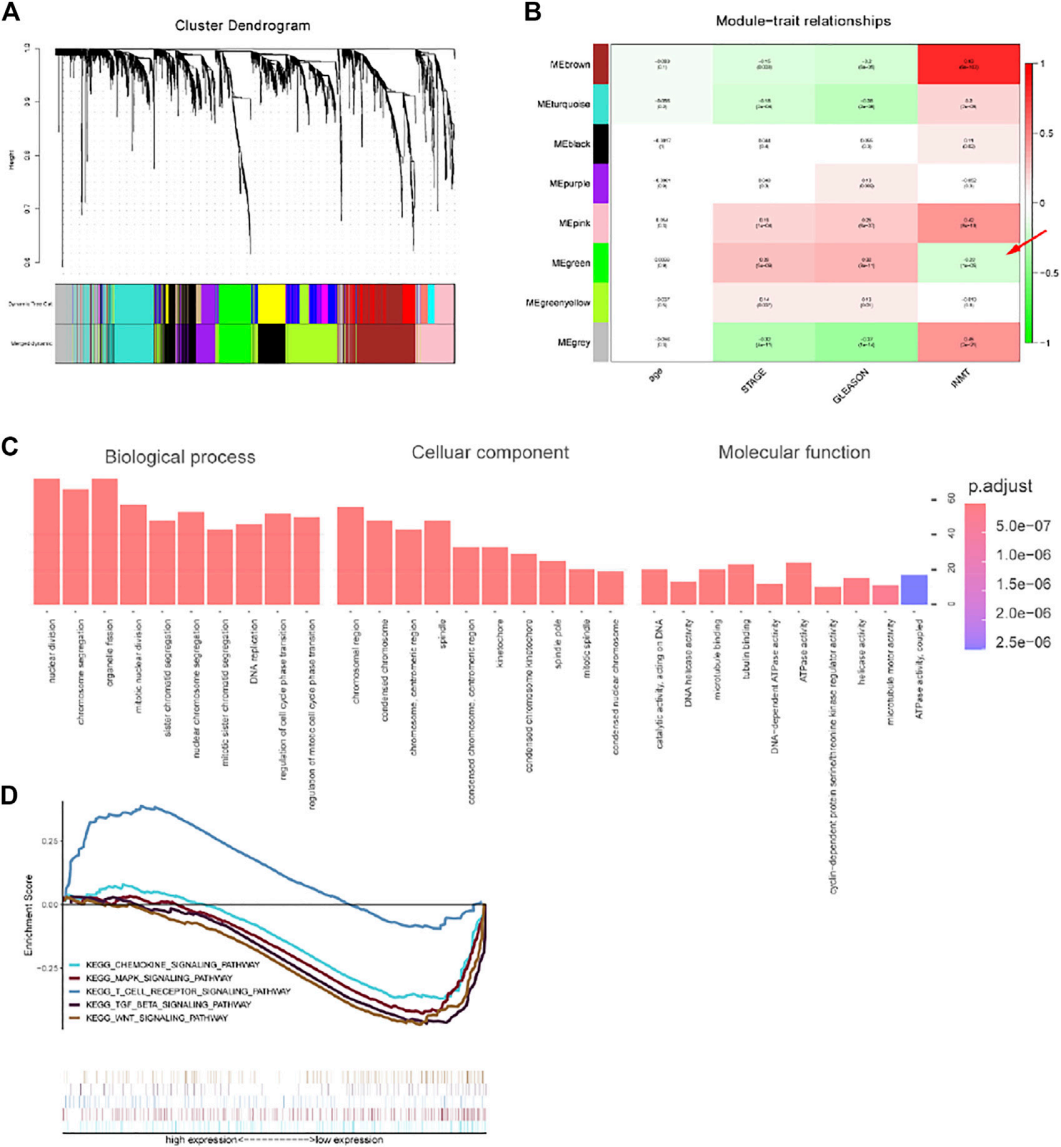
Results of weighted gene co-expression network analysis in TCGA-PRAD. **(A)** A hierarchical cluster tree was constructed, with each leaf representing a gene and each branch representing a co-expression module. A total of eight co-expression modules were generated. **(B)** Correlation coefficients between different factors and co-expression modules, where the brown (COR = 0.83; *p* < 0.001), gray (COR = 0.45; *p* < 0.001), and pink modules (COR = 0.42; *p* < 0.001) had a positive correlation with INMT, and the green module (COR = -0.22; *p* < 0.001) had a negative correlation with INMT. **(C)** The pathway enrichment analysis of the INMT co-expression genes in the green module. **(D)** The KEGG pathway annotation and enrichment analysis of the INMT co-expression genes in the green module.

To explore the biological process of INMT and construct its co-expression network, we constructed a co-expression module and performed a module-trait analysis based on the clinical characteristics of individuals with prostate cancer and the expression of INMT. The expression of INMT was positively linked with the brown (COR = 0.83; *p* < 0.001), gray (COR = 0.45; *p* < 0.001), and pink modules (COR = 0.42; *p* < 0.001), and negatively linked with the green module (COR = -0.22; *p* < 0.001) (Figures 3A,B). Through pathway enrichment analysis, we found that the co-expressed genes in the green module were drastically associated with biological processes consisting of nuclear division, chromosomal segregation, organelle fission, and mitotic nuclear division (Figure 3C). KEGG pathway annotation and enrichment analysis once again confirmed that the expression of INMT was closely related to MAPK, TGFβ, and Wnt signaling pathways. In addition, we also found that the co-expressed genes were associated with the chemokine and T cell receptor signaling pathways. We speculated that the INMT expression might shape the tumor microenvironment and immune cell infiltration (Figure 3D).

### Overexpressed INMT Inhibited Prostate Cancer Cell Proliferation and Apoptosis

**FIGURE 4:**
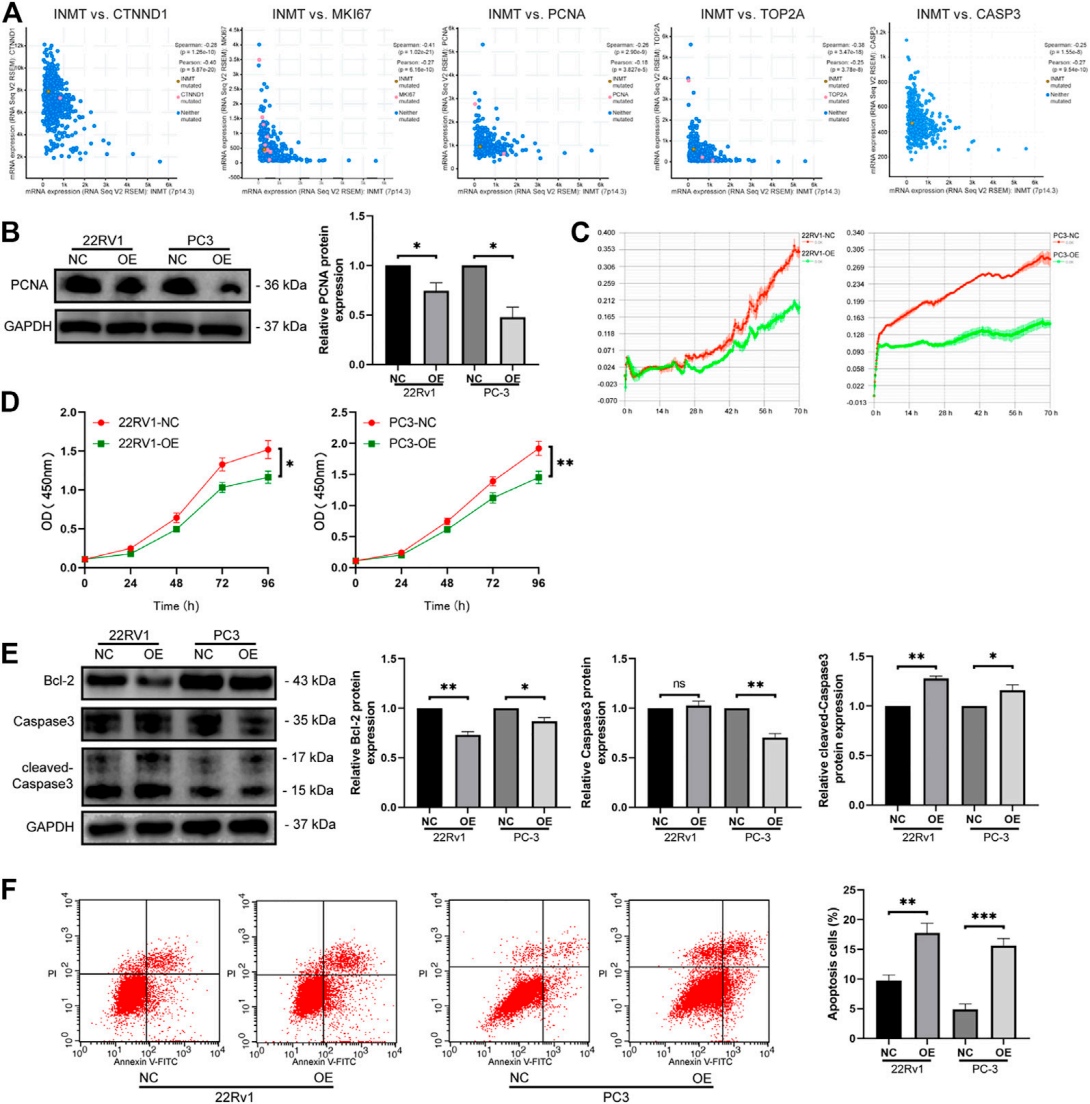
Overexpression of INMT inhibited cell activity and proliferation and promoted apoptosis. **(A)** Correlation between INMT and proliferation-related genes (*CTNND1*, *MKI67*, *PCNA*, *TOP2A*) and apoptosis-related gene. **(B)** PCNA protein levels were measured using western blotting. **(C)** Overexpression of INMT suppressed cell viability according to the RTCA assay. **(D)** The effect of INMT overexpression on cell proliferation was determined using the CCK-8 assay. **(E)** Bcl-2, caspase 3, and cleaved caspaase3 protein levels were measured using western blotting. **(F)** Apoptosis was measured using flow cytometry in 22Rv1 and PC-3 cells after INMT overexpression.

Through Pearson correlation analysis, we found that INMT was associated with the expression of many genes, including critical genes linked to cell proliferation along with apoptosis. Of these, the positive correlations with *CTNND1*, *MKi67*, *PCNA*, *TOP2A*, and *CASP3* were the most significant (Figure 4A, *p* < 0.001). Western blotting data confirmed that with the overexpression of INMT, the expression of proliferation-related protein PCNA was downregulated (Figure 4B). To verify the biological roles of INMT in prostate cancer, we used real-time cell analysis (RTCA) and the Cell Counting Kit 8 (CCK8) assays to determine the role of INMT in prostate cancer cell proliferation. We established that the proliferation of PC-3 and 22Rv1 cells decreased significantly after overexpression of INMT (Figures 4C,D). Western blotting data of apoptosis-linked factors illustrated that with the overexpression of INMT, the protein content of Bcl-2 decreased remarkably, while the protein content of cleaved-caspase three increased with the change of the total amount of caspase-3 (Figure 4E). Apoptosis assays of 22Rv1 and PC-3 cells demonstrated that the overexpression of INMT was remarkably linked to increased apoptosis (Figure 4F).

### Tumor Microenvironment and Immune Cells Infiltration

**FIGURE 5:**
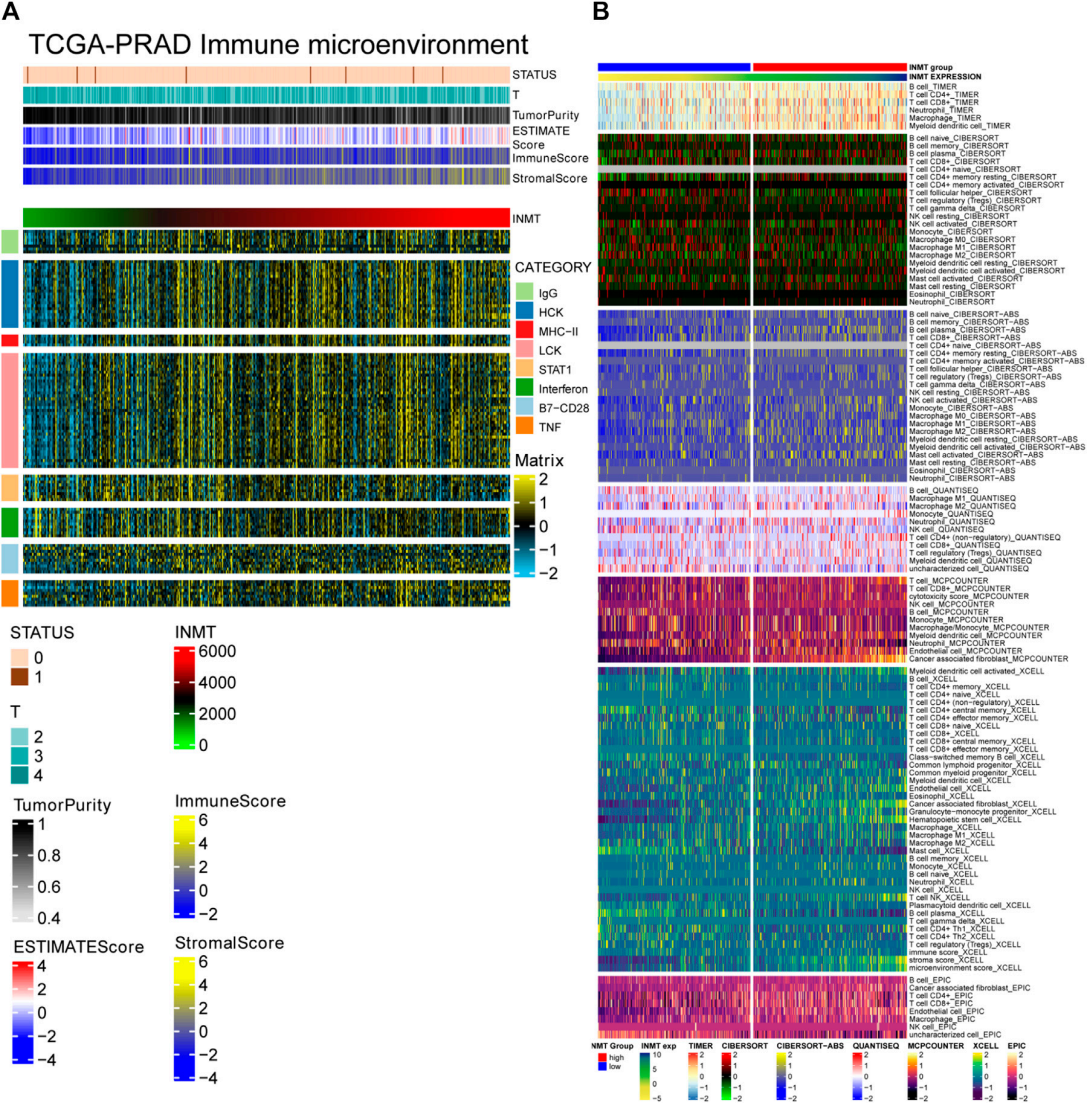
**(A)** Correlation between the expression level of INMT and tumor stage, tumor purity, ESTIMATE score, immune score, stormal score and immune response intensity (expression of eight immune-related genes). **(B)** The proportion of different immune cells in the two subgroups of INMT expression based on TIMER, CIBERPORT, CIBERPORT-ABS, QUANTISEQ, MCPcounter, XCELL and EPIC algorithms.

INMT is located in the Golgi apparatus and vesicles, mainly involved in protein processing and cell secretion. Tumor cells can change the tumor microenvironment through cell secretion. The complexity and diversity of immune cell infiltration in the tumor microenvironment critically impact tumor immunotherapy. The ESTIMATE algorithm was applied to calculate the correlation between the expression level of INMT and tumor stage, tumor purity, ESTIMATE score, immune score, stromal score, and immune response intensity (expression of eight immune-related genes). Heatmap showed that with the increase of INMT expression, tumor stage decreased, tumor purity decreased, ESTIMATE score increased, the immune score increased, the stromal score increased, and immune response intensity increased (Figure 5A). The proportion of different immune cells in the two subgroups of INMT expression was calculated based on TIMER, CIBERPORT, CIBERPORT-ABS, QUANTISEQ, MCPcounter, XCELL, and EPIC algorithms. In the subgroup with high expression of INMT, a variety of immune cells and tumor microenvironment components increased, such as B cell, T cell CD4^+^, T cell CD8^+^, macrophage, myoid dendritic cell, endothelial cells, and cancer-associated fibroblasts (Figure 5B).

Identification of potential therapeutic agents based on the expression of INMT in prostate cancer.

**FIGURE 6:**
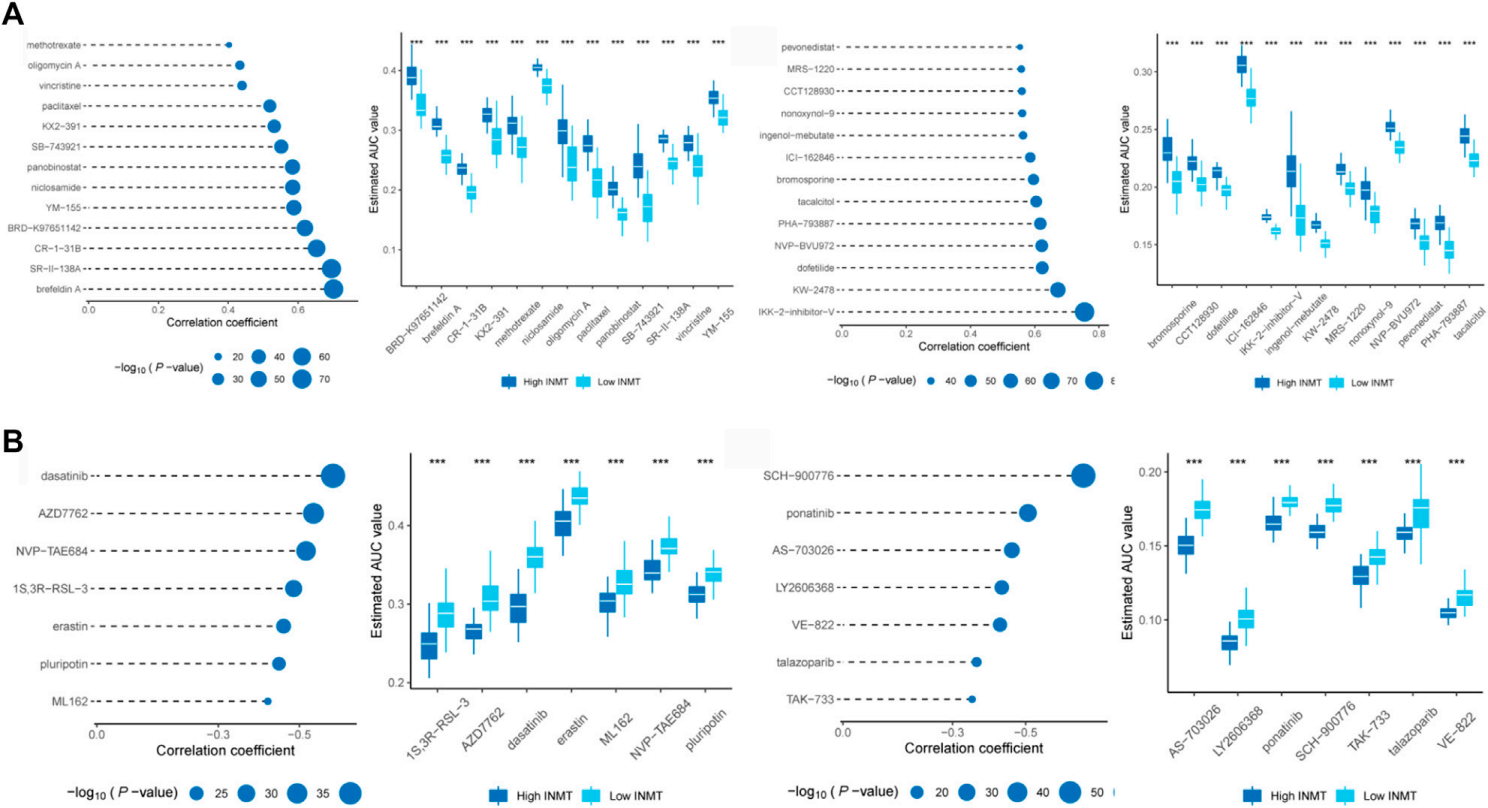
Identification of candidate agents with high drug sensitivity in patient groups with various INMT expression levels. Based on CTRP and PRISM datasets, **(A)** Spearman correlation analysis found that the AUC values of 26 compounds were positively correlated with the expression of INMT, **(B)** while the AUC values of 14 compounds were negatively correlated with the expression of INMT. Note that the lower the value on the Y-axis of the box plot, the higher the drug sensitivity.

According to the analysis results of PRISM (https://www.theprismlab.org) and CTRP2.0 (https://portals.broadinstitute.org/ctrp.v2.1/), the drug sensitivity area under the curve (AUC) values data were obtained and combined with cell line expression profile data. The potential therapeutic agents associated with the INMT expression subgroup in TCGA were predicted (log2FC > 0.10). Pearson correlation analysis was performed between AUC values and INMT subgroups, and compounds with positive (>0.4) and negative correlation coefficients (<-0.3) were selected. The AUC values of 26 compounds were positively correlated with the expression of INMT, while the AUC values of 14 compounds were negatively correlated with the expression of INMT. These findings suggest that prostate cancer patients with low INMT levels may demonstrate better drug sensitivity to these 26 compounds. Similarly, prostate cancer patients with elevated INMT levels may exhibit a high drug sensitivity to another 14 compounds (Figure 6).

## Discussion

INMT participates in detoxifying selenium compounds and regulates the tryptophan metabolic pathway by catalyzing the N-methylation of tryptamines and structure-related compounds (Kuehnelt et al., 2015). Studies suggested that the expression of INMT is downregulated in lung cancer, meningioma, and prostate cancer. Using pan-cancer analysis, we found that the different expression of INMT between cancer and adjacent non-malignant tissues was widespread in several tumors. The expression of INMT was significantly negatively correlated with lymph node metastasis, Gleason score, and PSA level, suggesting that INMT may be involved in the biological processes of prostate cancer proliferation, migration, and invasion. Through the construction of an INMT co-expression module and characteristic analysis, we found that genes co-expressed with INMT were closely associated with biological processes such as nuclear division, chromosome segregation, organelle fission, mitotic nuclear division, and regulation of cell cycle phase transition. These biological processes affect tumor cell proliferation, apoptosis, and genomic stability (Jefford and Irminger-Finger, 2006; Jeong and Seol, 2008; Santaguida and Amon, 2015). By analyzing the correlation between proliferation and apoptosis genes and proteins and *in vitro* experiments, we found that the expression of INMT affected the proliferation and apoptosis of prostate cancer cells.

Using pathway analysis, we found that, in addition to tryptophan metabolism and selenocompound metabolism, INMT was involved in many tumor signaling pathways, including the MAPK, TGFβ, and Wnt signaling pathways. The p38 MAPK signaling pathway is essential for the MAPK system (Wagner and Nebreda, 2009). It positively and negatively regulates prostate cancer cells’ growth, proliferation, and apoptosis (Wynne and Djakiew, 2010; Park et al., 2020). We found that overexpression of INMT activated the p38 MAPK signaling pathway, which was more significant in the hormone-sensitive prostate cancer 22Rv1 cell line. TGFβ is a polypeptide cytokine with many biological activities that regulate cell migration, adhesion, proliferation, and differentiation (Schmierer and Hill, 2007). The Smad-mediated TGFβ signaling pathway has dual activities in tumorigenesis. In early cancer cells, it inhibits cell cycle progression and induces apoptosis. In advanced tumors, carcinogenic mutations inactivate these tumor suppressor functions, inducing or enhancing epidermal-mesenchymal transition and invasion and metastasis of tumor cells (Ikushima and Miyazono, 2010; Colak and Ten Dijke, 2017). We found that INMT negatively regulated the TGFβ signaling pathway and was the most significant in a hormone-resistant prostate cancer cell line (PC-3). It has the most significant effect on Smad4 in the pathway. Wnt/β-catenin signaling is critical to the classical Wnt signaling pathway. Its activation affects prostate cell proliferation, differentiation, and invasion (Kypta and Waxman, 2012). This finding suggests that INMT might mediate the interaction of tumor signaling pathways or between metabolic and signaling pathways.

The microenvironment of prostate cancer includes blood vessels, immune cells, fibroblasts, and extracellular matrix. The changes of cell subsets and transcription levels lead to prostate cancer’s high heterogeneity and determine outcomes in prostate cancer (Chen et al., 2021; Thienger and Rubin, 2021). A study found that tumor microenvironment and tumor cells can interact and regulate one another through metabolites (Elia and Haigis, 2021). Studies showed that tryptophan metabolism regulates the immune microenvironment through several mechanisms associated with the occurrence and development of tumors (Liu and Zhai, 2021; Platten et al., 2021). We found that INMT, as a critical enzyme in tryptophan metabolism, is closely associated with the tumor microenvironment. In recent years, the tryptophan metabolizing enzyme aromatic hydrocarbon receptor pathway has become a research hotspot in tumor immunotherapy. A variety of metabolizing enzymes, including interleukin-4-induced-1, have become potential immunotherapeutic targets (Sadik et al., 2020). These findings suggest that INMT might be a metabolic checkpoint or immunotherapeutic target. By analyzing the sensitive drugs associated with INMT, we found that the sensitive drugs associated with low expression of INMT were mainly associated with tumor apoptosis. For example, brefeldin A, a common protein transport inhibitor, induces endoplasmic reticulum stress and apoptosis (Moon et al., 2012). An inhibitor of eukaryotic translation initiation factor 4A, CR-1–31B, has a synergistic effect on inhibiting the growth of tumor cells and inducing apoptosis in drug combination therapy (Kong et al., 2019). KW-2478 is a non-ansamycin Hsp90 inhibitor that induces tumor cell apoptosis by PARP lysis (Nakashima et al., 2010). There are relatively few sensitive drugs associated with the elevated expression of INMT. Dasatinib is a tyrosine kinase inhibitor of the variation of the Philadelphia chromosome and SRC gene. Its effectiveness in prostate cancer is still being explored. As an inhibitor of checkpoint kinase 1, SCH-900776 participates in the DNA damage-response pathway. The combined application with other antitumor drugs can enhance its anticancer effect.

In conclusion, INMT is expressed at a low level in prostate cancer and may affect the apoptosis and proliferation of prostate cancer cells through the MAPK, TGFβ, and Wnt signaling pathways. INMT may affect the occurrence and development of tumor cells by changing the tumor microenvironment and drug resistance mechanism in prostate cancer. The single-gene study of INMT enriches our understanding of its molecular functions. These findings provide a theoretical basis for studying the pathogenesis and treatment of prostate cancer, especially relating to INMT.

## Methods

### Ethics Statement

Approval of this research work was granted by the Ethics Committee of the First Hospital of China Medical University (No. [2018] 2018-190-2). Besides, tissue sample collection was as per the Declaration of Helsinki. The subjects granted written informed consent. The relevant guidelines and regulations were adhered to in implementing all the study methods.

### Matrix Source

The mRNA matrix and clinical data of prostate cancer were abstracted from the TCGA data resource (https://www.cancer.gov/) (Wang et al., 2016). Overall, 547 samples consisting of 495 prostate cancer samples and 52 non-malignant prostate samples were utilized. In addition, 36 prostate cancer tissue matrix samples coupled with their clinical data were abstracted from the GSE46602 data resource (a repository of GPL570 Affymetrix Human Genome U133 Plus 2.0 Array) (Mortensen et al., 2015). Drug sensitivity data were abstracted from the PRISM Repurposing resource (19Q4 December 2019 release, https://depmap.org/portal/prism/) and the Cancer Therapeutics Response Portal resource (CTRP v.2.0 October 2015 release, https://portals.broadinstitute.org/ctrp).

### Pathway Analysis

Up to date, there has been limited research on INMT. GSEA (Gene Set Enrichment Analysis) was employed to determine the cascades linked to INMT in prostate cancer (Subramanian et al., 2005). The GSEA approach determines if a set of interest genes remarkably differ in two biological states. GSEA was utilized to elucidate the functional cascades linked to INMT RNA expression, stratifying based on the median of the RNA expression of INMT. We carried out a cross-verification assessment between GSE46602 and TCGA-PRAD using a single cohort exhibiting a high false positive result.

### INMT Co-expression mRNA Analysis

It was established that INMT was remarkably associated with the TGFβ/Smad signaling cascade. Research evidence documents that the TGFβ/Smad signaling cascade is indispensable in numerous biological processes. Hence, Gene Ontology (GO) assessment (http://geneontology.org/) was adopted to elucidate the roles of protein-encoding genes of INMT co-expression (Ashburner et al., 2000). The weighted gene co-expression network approach was adopted to uncover INMT co-expression factors via developing a scale-free network (Langfelder and Horvath, 2008). The minimum module gene numbers were defined as 30. The module harboring a unique relationship with INMT was identified via establishing an association heatmap between INMT and the module. Pearson correlation coefficients of >0.4 were employed to abstract the protein-encoding genes. The GO assessment of these genes uncovered the most remarkable biological roles and the molecular functions of INMT. Screening was not directly based on the IMNT Pearson correlation coefficient; instead, its co-expression module was first established before the screening. This screening approach presumes that co-expression modules exhibit comparable biological features. The most remarkable co-expression module is determined via this approach, uncovering the most significant biological role.

### Correlations of Immune Cell Fractions

There is an INMT-triggered regulation of the immune response. Hence, we explore the related immune processes by assessing prostate cancer tissues’ immune cell contents. The CIBERSORT algorithm was adopted to assess immune cell fractions based on the expression matrixes of bulk tissue genes. LM22 constitutes a matrix of gene signature, which defines 22 sub-kinds of immune cells abstracted from a web resource (https://cibersort.stanford.edu/).

### Collection of Clinical Specimens

Radical prostatectomy was conducted on individuals with prostate cancer beginning January 2020 to December 2020 as per the EAU Guidelines on Prostate Cancer. Thirty paired samples of prostate cancer tissues and adjacent non-cancerous prostate tissue samples (>1 cm from the tumor) were acquired at the First Hospital of China Medical University (Shenyang, China). None of the subjects had been treated with chemotherapy, endocrine therapy, or radiotherapy prior to the radical prostatectomy. Histological assessment of all the was done. The prostate cancer tissue samples exhibited high-density cancer foci; however, the adjacent non-cancerous prostate tissues did not harbor cancer foci. All the samples were maintained at −80°C prior to analyses.

### Reagents and Cell Lines

STR-certified PC-3 and 22Rv1 human prostate cancer cells were supplied by the National Collection of Authenticated Cell Cultures (Shanghai, China). Detection of *Mycoplasma* in the growth medium was done via PCR, and the cell passage time did not exceed 6 months. All the study cells were inoculated in RPMI 1640 (HyClone, United States) added 10% FBS (Gibco) under 37°C and 5% CO_2_ conditions.

### Development of INMT Over-expressing Cell Lines

PC-3 and 22Rv1 cells were planted in six-well or other plates and left to grow for 24 h prior to transfection. Genechem (Shanghai, Genechem) designed and synthesized the INMT overexpression lentiviral vector and the respective negative control lentiviral vector. Transfection of the cell lines was carried out as documented in the manufacturer’s manual. The establishment of stable negative control and INMT overexpression cell lines was done in a puromycin-containing culture.

### RNA Isolation and Real-Time PCR

RNA from prostate cancer cells was purified using the TRIzol reagent (Invitrogen) as described by the manufacturer. PrimeScript^TM^ RT reagent kit (Takara, Japan) was used for reverse transcription, and SYBR premix ExTaq™ kit (Takara, Japan) was adopted to conduct RT-PCR. GAPDH served as the internal reference. Relative gene expressions were calculated using the 2^−ΔΔCT^ approach. Primer sequences were as follows: INMT (forward primer, TGG​AGA​AAG​AGG​AGG​TGG​AGC​AG; reverse primer, GGC​AGC​ATT​GGT​GAC​AGA​GTA​GC) and GAPDH (forward primer, GGA​GCG​AGA​TCC​CTC​CAA​AAT; reverse primer, GGC​TGT​TGT​CAT​ACT​TCT​CAT​GG).

### Western Blot Analysis

The radioimmunoprecipitation assay (RIPA) lysis buffer harboring with protease and phosphatase suppressors was utilized to purify proteins from tissues and cell lines. The bicinchoninic acid (BCA) assay was adopted to quantitate the proteins (Beyotime Institute of Biotechnology). After that, fractionation of the proteins (40 µg/lane) was done with the 10% SDS-PAGE gel (140 V, 60 min), then transfer-embedded onto PVDF membranes (340 mA, 90 min). Afterward, the membranes were blocked using 5% fat-free milk in Tris-buffered saline with 1% Tween 20 (TBS-T) (37°C, 1 h), followed by 4°C overnight incubation with primary antibodies. After washing with TBS-T, the membranes were incubated with the appropriate HRP-labeled secondary antibodies (37°C, 1 h). After rewashing the membranes, the blots were displayed on the MicroChemi Chemiluminescent Imaging System (DNR Bio-Imaging Systems, Mahale HaHamisha, Jerusalem, Israel) using the ECL reagents (Transgen Biotechnology, Beijing, China). ImageJ 1.52p software (Wayne Rasband, National Institutes of Health, United States) was used to calculate the gray value of each band, and statistical analysis was carried out according to the ratio of target protein/GAPDH. The primary antibodies consisted of INMT (Genetex, GTX119115), phospho-p38 MAPK (Cell Signaling Technology, 4511T), p38 MAPK (Cell Signaling Technology, 8690T), TGFβ (Abcam, ab215715), Smad2 (Cell Signaling Technology, 5339S), SMAD4 (Cell Signaling Technology, 46535S), beta-catenin (Proteintech, 51067-2-AP), cyclin D1 (Cell Signaling Technology, 55506S), PCNA (Cell Signaling Technology, 2586S), Bcl-2 (Cell Signaling Technology, 15071S), caspase-3 (Cell Signaling Technology, 9662S), and GAPDH (Cell Signaling Technology, 5174S). They were used according to the manufacturer’s protocols.

## RTCA

5,000 of 22Rv1 and PC-3 cells per well were inoculated in E-plates under 37°C and 5% CO_2_ conditions for 72 h. RTCA xCELLigence S16 automatically recorded the cell growth curve.

### CCK-8 Assay

As documented by the manufacturer (Bimake, United States), CCK-8 solution was introduced to every well of 96-well plate at 0.5 mg/ml. A plate reader (Model 680; Bio-Rad Laboratories) measured the absorbance of cells at 450 nm.

### Cell Apoptosis Analyses

An Annexin V-FITC Apoptosis Detection Kit (Beyotime) was adopted for apoptotic analyses. As documented by the manufacturer, staining of cells was done using annexin V-FITC and PI solution (15 min at room temperature (RT)). A FACSCalibur Flow Cytometer was adopted to assess apoptosis.

### Statistical Analysis

Data analyses were implemented in the GraphPad Prism v.8.0 software (GraphPad Inc., United States). Data are given as mean ± SD for *n* = 3. A student’s t-test was adopted to explore group differences, with *p* < 0.05 signifying statistical significance. Pearson coefficients >0.4 signified significance. The R v. 3.6.3 software was adopted for R package statistical analyses.

## Data Availability

Publicly available datasets were analyzed in this study. This data can be found here: TCGA-PRAD: http://cancergenome.nih.gov/ GSE46602: http://www.ncbi.nlm.nih.gov/geo/.

## Abbreviations

CCK-8, Cell Counting Kit eight assay; GO, Gene Ontology; INMT, indolethylamine-N-methyltransferase; NC, negative control cell lines; OE, INMT overexpression cell lines; PSA, prostate-specific antigen; RTCA, Real-Time Cell Analysis; TCGA, The Cancer Genome Atlas; WGCNA, weighted gene co-expression network.
