# Supplementary material for: Indolethylamine-N-Methyltransferase Inhibits Proliferation and Promotes Apoptosis of Human Prostate Cancer Cells: A Mechanistic Exploration
Source: Front Cell Dev Biol. 2022 Feb 17;10:805402. doi: 10.3389/fcell.2022.805402 (PMC8891133; doi:10.3389/fcell.2022.805402)
Supplement: Supplementary file 1 [file Table1.DOCX]

**Supplementary Table 1 Clinicopathological data of 30 patients with prostate cancer**

| case | age (years) | preoperative PSA (ng/ml) | Gleason score | TNM stage (AJCC, 2017) | number of positive lymph nodes / lymph nodes | positive surgical margin | prostate volume (ml) | preoperative endocrine therapy |
| --- | --- | --- | --- | --- | --- | --- | --- | --- |
| 1 | 72 | 31.1 | T3aN1M0 | 4+5=9 | L (0/3); R (13/14) | Yes | 26.56 | No |
| 2 | 49 | 14.9 | T4N1M0 | 5+4=9 | L (0/7); R (1/9) | Yes | 32.09 | No |
| 3 | 64 | 9.2 | T2cN0M0 | 3+5=8 | L (0/4); R (0/4) | No | 31.60 | No |
| 4 | 61 | 15.9 | T3aN0M0 | 4+3=7 | L (0/4); R (0/6) | Yes | 16.25 | No |
| 5 | 68 | 11.9 | T2cN0M0 | 5+3=8 | L (0/9); R (0/3) | No | 54.93 | No |
| 6 | 67 | 10.6 | T1cN0M0 | 3+3=6 | L (0/17); R (0/10) | No | 111.65 | No |
| 7 | 65 | 14.7 | T2cN0M0 | 5+3=8 | L (0/6); R (0/4) | No | 55.92 | No |
| 8 | 69 | 5.5 | T2cN0M0 | 3+3=6 | L (0/4); R (0/2) | No | 53.66 | No |
| 9 | 62 | 19.5 | T2cN0M0 | 3+4=7 | L (0/3); R (0/2) | No | 38.61 | No |
| 10 | 75 | 14 | T2aN0M0 | 3+4=7 | L (0/4); R (0/4) | No | 55.95 | No |
| 11 | 56 | 17.6 | T1cN0M0 | 3+4=7 | L (0/5); R (0/4) | No | 21.41 | No |
| 12 | 74 | 10.7 | T2cN0M0 | 3+4=7 | L (0/3); R (0/4) | No | 27.23 | No |
| 13 | 55 | 6.4 | T2cN0M0 | 4+3=7 | L (0/4); R (0/2) | No | 14.31 | No |
| 14 | 72 | 7.0 | T1cN0M0 | 3+3=6 | L (0/4); R (0/4) | No | 19.14 | No |
| 15 | 64 | 9.2 | T2cN0M0 | 3+5=8 | L (0/4); R (0/4) | No | 31.60 | No |
| 16 | 55 | 23 | T2cN0M0 | 4+3=7 | L (0/5); R (0/15) | No | 38.69 | No |
| 17 | 62 | 7.4 | T2cN0M0 | 5+4=9 | L (0/6); R (0/2) | No | 29.65 | No |
| 18 | 69 | 10.0 | T2aN0M0 | 4+3=7 | L (0/6); R (0/1) | No | 54.80 | No |
| 19 | 55 | 8.0 | T2cN0M0 | 3+4=7 | L (0/2); R (0/1) | No | 23.40 | No |
| 20 | 75 | 16.1 | T2cN0M0 | 3+4=7 | L (0/6); R (0/4) | No | 28.61 | No |
| 21 | 59 | 22.7 | T2cN0M0 | 3+4=7 | L (0/5); R (0/4) | No | 41.57 | No |
| 22 | 67 | 10.8 | T2cN0M0 | 3+4=7 | L (0/1); R (0/2) | No | 17.01 | No |
| 23 | 48 | 27.9 | T2cN0M0 | 3+4=7 | L (0/8); R (0/5) | No | 39.04 | No |
| 24 | 69 | 39.2 | T2cN0M0 | 3+4=7 | L (0/6); R (0/2) | No | 48.63 | No |
| 25 | 69 | 12.1 | T2cN0M0 | 4+3=0 | L (0/2); R (0/5) | No | 37.96 | No |
| 26 | 62 | 23.9 | T2bN0M0 | 4+5=9 | L (0/9); R (0/9) | No | 30.95 | No |
| 27 | 62 | 37.8 | T3aN0M0 | 4+3=7 | L (0/13); R (0/12) | Yes | 36.24 | No |
| 28 | 62 | 20.6 | T2cN1M0 | 5+4=9 | L (0/2); R (2/13) | No | 10.50 | No |
| 29 | 67 | 21.4 | T2cN0M0 | 3+3=6 | L (0/11); R (0/12) | No | 45.86 | No |
| 30 | 71 | 12.7 | T2bN0M0 | 3+4=7 | L (0/10); R (0/4) | No | 53.52 | No |
